# A case of cardiac angiosarcoma and pulmonary tuberculosis; diagnostic challenges and review of literature

**DOI:** 10.22088/cjim.12.0.500

**Published:** 2021

**Authors:** Roghayeh Pourkia, Ghasem Faghanzadeh Ganji, Mohsen Vakili Sadeghi, Naghmeh Ziaie, Nahid Rezaeian, Faezeh Dehghani-Tafti, Hossein Ghorbani, Mohammad Mostafa Ansari Ramandi

**Affiliations:** 1 Department of Cardiology, Babol University of Medical Sciences, Babol, Iran; 2 Department of Cardiac Surgery, Ayatollah Rouhani Hospital, Babol University of Medical Sciences, Babol, Iran; 3 Cancer Research Center, Babol University of Medical Sciences, Babol, Iran; 4 Department of Cardiology, Babol University of Medical Sciences, Babol, Iran; 5 Yazd Cardiovascular Research Center, Shahid Sadoughi University of Medical Sciences, Yazd, Iran; 6 Department of Pathology, Babol University of Medical Sciences, Babol, Iran; 7 Cardiovascular Diseases Research Center, Birjand University of Medical Sciences, Birjand, Iran

**Keywords:** Heart neoplasms, Tuberculosis, Prognosis, Magnetic resonance imaging

## Abstract

**Background:**

Patient symptoms and primary investigational methods may be misleading at some points in patient management and can consume a lot of time. Sarcomas are rare malignancies and contribute 1% of all cancers of adult.

**Case Presentation:**

A rare case of primary cardiac angiosarcoma is presented, who was first treated because of lung tuberculosis and then with only slight improvement in symptoms, further investigations were done showing right ventricular enlargement and pericardial effusion. Eventually, after ruling out pulmonary embolism and constrictive pericarditis, investigations lead to the diagnosis of primary cardiac angiosarcoma. The patient went under surgery to remove the tumor but he still had residual mass left, leading to chemotherapy and then radiotherapy. Although the tumor has a poor prognosis, our patient has managed to survive a year by now and is doing good for 6 months after radiotherapy.

**Conclusion:**

The case describes the importance of having in mind different differential diagnosis in managing patients and the role of multi-modality imaging in guiding diagnosis and treatment.

Reaching the correct diagnosis may be difficult and time-consuming, especially when dealing with rare diseases. Symptoms such as dyspnea, cough, and easy fatigability have many differential diagnoses, making it hard to find the actual diagnosis. Other than respiratory diseases and cancers which can cause these symptoms, primary cardiac tumors can also present with the same symptoms. Sarcomas are rare malignancies and contribute 1% of all cancers of adult. More than 50 histologic subtypes of soft tissue sarcomas were diagnosed (1). Primary cardiac angiosarcoma is a highly aggressive tumor of the heart which usually originates from the right atrium. It can present itself with nonspecific symptoms, such as cough, dyspnea, and heart failure (2). Tuberculosis, however, can present with other symptoms as well such as night sweats, unexplained weight loss and chest pain. Long-term cough is a common finding in patients diagnosed with tuberculosis (3). Early stages of cardiac angiosarcoma may have a better prognosis, while later stages have a poor prognosis. In the early stages, radical resection with adjuvant radiotherapy is the standard therapy (4), while for advanced stages, chemotherapy, radiotherapy, and surgery are preferred together (5). We present a 40-year-old male with primary cardiac angiosarcoma who was under treatment for diagnosed tuberculosis 6 months before diagnosing the tumor. Considering the delay in diagnosis, our case seems to have well responded to the combined treatment strategy.

## Case presentation

**Figure 1:**
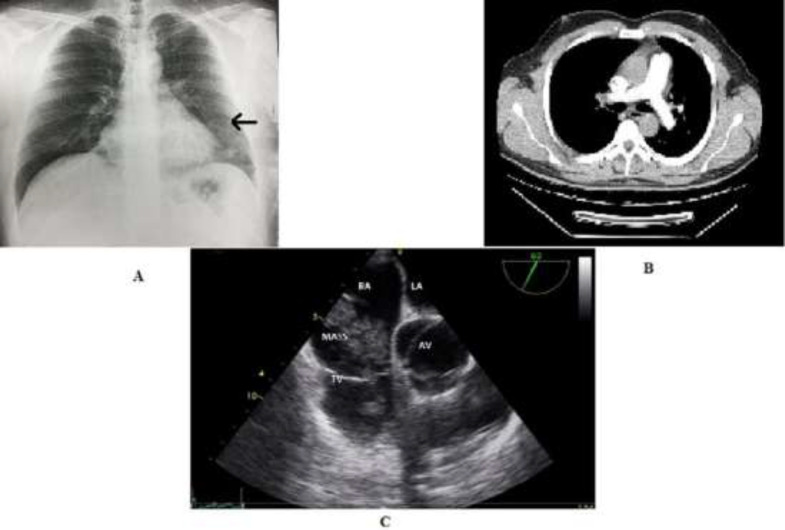
A, chest x-ray showing possible bronchiectasis and left lung lower lobe collapse (black arrow). B, pulmonary CTA showing no evidence of pulmonary embolism. C, transesophageal echocardiography showing the mass protruding through tricuspid valve

A 40-year-old male is presented with dyspnea and cough 2 months before workup in another center. He came to our center in Babol University of Medical Sciences in April 2019, 6 months after his initial workup in the primary center. In the initial evaluation, the patient was a middle-aged man without a history of any disease or family history of a disease. He was a smoker and did not drink alcohol. On first physical examination, he had crackles in the base of the left lung with other findings being unremarkable. In para-clinical evaluation, on chest x-ray (figure 1A) and chest computed tomography (CT) scan, there was evidence of bronchiectasis and left lung lower lobe collapse. Coming from the Middle East, with all the symptoms described, the first differential diagnosis for the patient was tuberculosis (TB). Sputum evaluation for mycobacterium tuberculosis was negative and bronchoscopy and bronchoalveolar lavage were done for the patient and the pathology revealed positive Ziehl-Neelsen staining for TB but negative QuantiFERON. Considering the clinical setting and high probability of tuberculosis, treatment with anti-TB medications was started for the patient.

After 2 months of treatment, although his symptoms had improved a bit, he reported back to the hospital with increased dyspnea and weakness and easy fatigability. On physical examination, he had muffled heart sounds and so echocardiography was done for the patient, which revealed mild pericardial effusion (PE) and normal left ventricle (LV) and right ventricle (RV) size and function. Anti-TB medications were continued and anti-inflammatory drugs added. His symptoms did not improve over the following 2 months and he had to be admitted again with worsening dyspnea. On evaluation, he had mild to moderate PE and mild RV enlargement. There was no evidence for constrictive pericarditis on echocardiography. D-dimer was requested for the patient which was reported to be high. Pulmonary computed tomography angiography was done for the patient which showed no evidence of pulmonary embolism (figure 1B). Previous medications were continued for the patient and the patient was discharged. After another 2 months of treatment and near the end of his TB treatment, he was referred to our center due to respiratory distress and dyspnea. On physical examination, there was a diastolic murmur at the left lower sternal border and crackles at the base of the left lung. Other than his respiratory rate which was 24/min, his other vital signs were within normal limits. Trans-thoracic echocardiography was done for the patient which showed mild RV enlargement and dysfunction, dilated inferior vena cava without collapse and mild pulmonary artery hypertension. There was thick pericardium with small PE and compressive effect on tricuspid valve with probable extracardiac source which had resulted in a significant diastolic pressure gradient. A large sized mobile mass was also seen in RV outflow tract.

**Figure 2:**
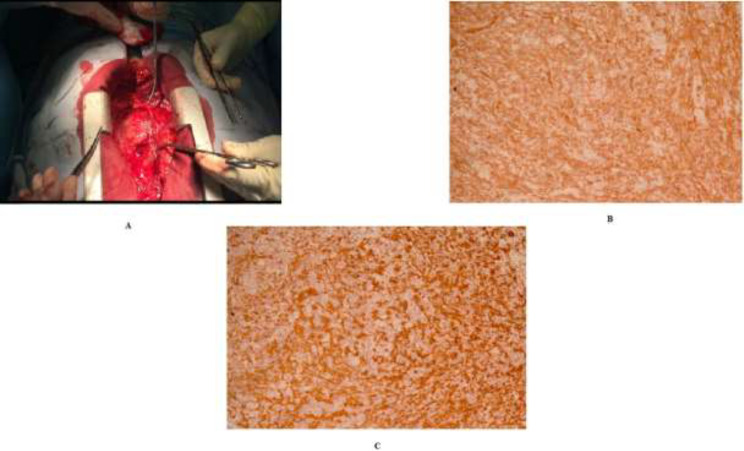
A, Macroscopy of the tissue during surgery. B, Immunohistochemistry showing CD 31 staining. C, Immunohistochemistry showing CD 34 staining

Transesophageal echocardiography was done for the patient which showed a very large size multilobulated nonhomogenous mass in anterolateral side of right atrium (RA) attached to the pericardium. One lobe of it was perforating the RA wall and protruding to RV outflow tract from tricuspid valve (figure 1C and supplementary video file 1). The patient had severe symptoms and so surgical consult was done for the patient to remove the mass (figure 2A). During surgery, after sternotomy, opening the pericardium was difficult because of the adhesions present. There was a large inflammatory mass without clear borders attached to the heart with especial attachment to the RA. It was removed to maximum extent and due to increasing blood oozing, the surgery was interrupted, mediastinal space temporarily packed and the patient was transferred to intensive care unit. After correcting the underlying coagulopathy, and repeating TEE, the patient was transferred to operating room and he was put on cardiopulmonary bypass. RA was surgically opened and there was fragile mass without capsule attached to the RA wall. The mass was excised to the maximum extent and after closing the chest wall, he was transferred to the ICU. On pathological evaluation, there was neoplastic tissue composed of fasciculated spindle cells being highly CD31 positive (figure 2B) and CD 34 positive with high Ki67 proliferation index (figure 2C). The final pathology report was in favor of high-grade angiosarcoma with massive necrosis. Abdominal and lung CT scan and whole-body bone scan was done for the patient which showed no evidence of metastasis.

**Figure 3:**
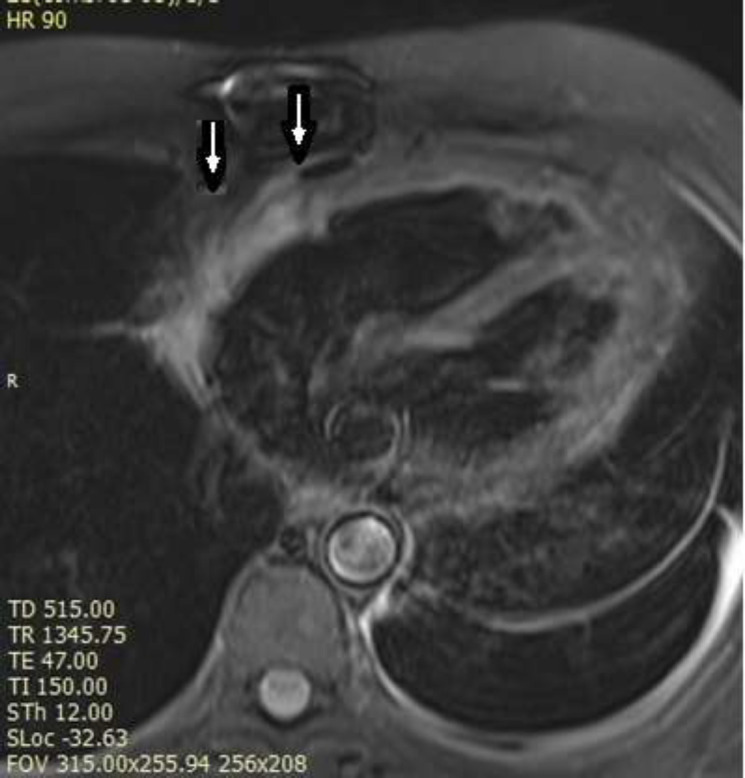
T2 Weighted STIR image shows high signal intensity mass at lateral part of RA wall (white arrows).

After the surgery, transesophageal echocardiography showed some residual mass in the RA, for which chemotherapy with docetaxel and gemcitabine were started for the patient. After six cycles of chemotherapy, the cardiac mass in the RA regressed and the cardiac function improved. To see if there is extracardiac tumor present or not, cardiac magnetic resonance imaging (CMR) was done for the patient. CMR showed a non-homogenous mass around RA and RA appendage with a size of 64x14mm (figure 3 and supplementary video file 2). There was mild pericardial thickening without hemodynamic evidence for constrictive pericarditis. Due to the residual mass, the multidisciplinary team meeting decided the patient undergo radiotherapy and close monitoring. After 25 sessions of radiotherapy, the patient is in good condition with no sign or symptoms. He has been symptom-free more than 6 months now. 

## Discussion

Primary cardiac neoplasms have a low incidence of 0.0001% to 0.030%, out of which one fourth are malignant with cardiac angiosarcoma being the most common pathology (1). Cardiac angiosarcomas are usually present in the right atrium and most commonly metastases to the bones and lungs (6). Although CMR, transthoracic echocardiography, CT scanning, and positron emission tomography-CT have been used for diagnosing the condition, biopsy and pathology are needed to confirm the diagnosis (7-8). Due to their invasive behavior, the early diagnosis of disease is important for better survivals. Usually, the tumor is missed because of its rare incidence and non-specific symptoms, which makes its treatment difficult in later stages of the disease. The first-line treatment in these patients is surgical resection but as in many cases, patients are diagnosed in later stages which may not allow the use of this treatment strategy. Other than surgery, chemotherapeutic agents have also been used and in some cases have been promising (4). 

In a case described by Sleszycka et al., the patient has mycobacterium tuberculosis complex genetic material in his tumor tissue and it is proposed that tuberculosis can be deliberated as a risk factor for angiosarcoma. They also report a delay in treatment because of diagnostic challenges (9). 

Kurian et al. also faced diagnostic challenges in a young man presenting with shortness of breath. He was first diagnosed as viral pericarditis and with further evaluation after eight weeks, the patient had increased size in the mass and was eventually diagnosed with primary cardiac angiosarcoma (10). 

In another case described by Suderman et al., the patient presents with shortness of breath and tachycardia and CMR is used as an initial work-up which showed PE and the mass adjacent to the esophagus. Fortunately, their patient also had a long progression-free survival period by using chemotherapy and radiotherapy concurrently (5). This brings up the issue of thinking of combined treatment strategies for improving outcomes in these patients. Considering the costs of CMR, it may seem beneficial to use a simpler, less expensive way such as echocardiography for initial work-up, but as in our case, there may be difficulties in identifying the mass if the operator does not pay much attention while evaluating the RA and the RV. Other than the mentioned pitfall, in our case, the patient had tuberculosis and most symptoms were attributed to the former diagnosis, which left angiosarcoma undiagnosed till almost TB treatment was completed, delaying the start of his treatment. Our case also had a positive high D dimer test, which returned to near normal levels in about 2 weeks after surgery, meaning that the test could be attributed to the malignancy itself. Although our patient had some delay in being diagnosed but fortunately responded well to surgery, chemotherapy and radiotherapy. There have been cases reported to have shorter survivals, but it is believed that after radical resection, targeted therapy, adjuvant chemotherapy and radiotherapy in a multidisciplinary approach can prolong survival to even 33 months (2). 

In conclusion although primary cardiac angiosarcoma is rare, having it in mind in the differential diagnosis is important, to not lose the treatment opportunities and better survivals. A multidisciplinary team is needed to manage difficult cases of patients reporting with increasing symptoms, who are not responding to primary diagnosis treatment plan. Multiple imaging methods should be used to guide treatment and help in patient management. It is always good to consider other conditions which may have the same symptoms, to not miss any other diseases which may have happened concurrently. 

## References

[1] Amankwah EK, Conley AP, Reed DR (2013). Epidemiology and therapies for metastatic sarcoma. Clin Epidemiol.

[2] Zhang R, Li L, Li X (2017). Primary cardiac angiosarcoma: A case report. Medicine.

[3] Lee S, Lau L, Lim K (2019). The presence of cough and tuberculosis: active case finding outcomes in the Philippines. Tuberc Res Treat.

[4] Patel SD, Peterson A, Bartczak A (2014). Primary cardiac angiosarcoma- a review. Med Sci Monit.

[5] Suderman D, Cooke A, Wong R, Klein J (2011). Treatment of cardiac angiosarcoma with radiation and docetaxel: a case report with partial response and prolonged stable disease. J Thorac Oncol.

[6] Yu JF, Cui H, Ji GM (2019). Clinical and imaging manifestations of primary cardiac angiosarcoma. BMC Med Imaging.

[7] Campanile A, Tavazzi G, Alam MH, Paul R, Price S (2016). An unexpected finding in an asymptomatic patient with atrial fibrillation: cardiac angiosarcoma. Lancet.

[8] Rahbar K, Seifarth H, Schäfers M (2012). Differentiation of malignant and benign cardiac tumors using 18F-FDG PET/CT. J Nucl Med.

[9] Sleszycka JA, Sioma A, Mazurkiewicz L (2013). Cardiac angiosarcoma with the presence of mycobacterium tuberculosis complex genetic material. Eur Heart J.

[10] Kurian KC, Weisshaar D, Parekh H (2006). Primary cardiac angiosarcoma: case report and review of the literature. Cardiovasc Pathol.

